# Traditional healers’ knowledge and infection control practices related to HIV in Bukavu City, Democratic Republic of the Congo

**DOI:** 10.1186/s12889-024-18941-9

**Published:** 2024-05-27

**Authors:** Célestin Kyambikwa Bisangamo, Nessrin Ahmed El-Nimr, Patrick Milabyo Kyamusugulwa, Iman Mohamed Helmy Wahdan, Zahira Metwally Gad

**Affiliations:** 1 Department of Public Health, Bukavu High Institute of Medical Techniques (ISTM-Bukavu), Bukavu, Democratic Republic of the Congo; 2 Department of Epidemiology, High Institute of Public Health, Alexandria University, Alexandria, Egypt

**Keywords:** Traditional healers, HIV/AIDS, HIV knowledge, Infection control practices, Bukavu

## Abstract

**Background:**

Patients with HIV consult traditional healers (THs). These THs can both delay care for people living with HIV (PLHIV) and transmit HIV through poor infection control practices. The main objective of this study was to evaluate knowledge and practices of THs regarding HIV in Bukavu.

**Methods:**

A cross-sectional study using quantitative approach was carried out among 71 THs in Bukavu City. The collected data included the following topics: personal and socio-demographic characteristics, HIV knowledge, and infection control practices. Descriptive statistics, independent-samples T-test or F-test, and multiple linear regression were used to analyze the data with a p-value < 0.05.

**Results:**

The THs’ mean age was 49.2 ± 11.2 years, and the majority were aged 40 to < 60 years. Males constituted 88.7% of THs with a male-to-female ratio of 7.9. In general, 47.9% of study participants had poor knowledge about HIV/AIDS infection, 45.1% of them had fair knowledge, and only 7.0% had good knowledge. Overall, 43.7% of THs had poor infection control practices, 52.1% of THs had fair practices, and only 4.2% of participants had good practices. Results of multiple linear regression analysis revealed that none of the personal and demographic variables studied were significant predictors of their knowledge about HIV/AIDS (*p* > 0.05). In terms of practices, two variables were significant predictors of infection control practices: living in Ibanda and receiving training in taking care of HIV/AIDS.

**Conclusion and recommendations:**

: The study revealed that THs’ knowledge about HIV infection was insufficient and that they had poor infection control practices. Formal standardized training on HIV infection should be organized for all THs so that they can always refer their patients to modern, reliable antiretroviral therapy (ART) clinics and reduce the risk of occupational exposure in their practices. Although PPE’s assistance for THs is required in terms of protective measures, the province health authority must also oversee infection control procedures at THs’ offices.

**Supplementary Information:**

The online version contains supplementary material available at 10.1186/s12889-024-18941-9.

## Background

In 2020, the Democratic Republic of the Congo (DRC) had 510,000 people living with human immunodeficiency virus (PLHIV), including nearly 20,000 newly infected people [1]. Despite significant progress in the fight against HIV, the epidemic continues to significantly impact public health in all regions [2]. In the DRC, HIV infection is a deadly threat, with less than 60% of PLHIV having access to ART due to limited supply, lack of information and preventive services, stigma, and drug costs [1]. HIV prevalence has been reported at 1.2% in Bukavu, the capital city of the South Kivu province in Eastern DRC [3].

Early HIV diagnosis and prompt treatment are critical for improving HIV treatment outcomes [4], lowering the cost of medical care [5], and significantly impacting disease prevention [6–9]. 

Although the DRC has a ratio of 10.6 physicians per 100,000 people, there is a significant disparity due to the concentration of the majority of these physicians in urban areas [10, 11]. In sub-Saharan Africa, nearly 80% of the black African population regularly receive healthcare services from THs due to both the accessibility and acceptability of their services [12–14]. The TH is someone who is acknowledged by the community in which they live as qualified to provide health care through the use of plant, animal, and mineral substances as well as certain other methods based on their social, cultural, and religious backgrounds as well as the prevalent knowledge, attitudes, and beliefs regarding social, mental, and physical well-being as well as the causes of disease and disability in the community [11]. Several studies have reported that the majority of patients with HIV use traditional medicine concurrently with modern medicine [15–17]. It is estimated that between 60 and 80% of HIV patients use traditional medicine [18]. 

Traditional Healers can both delay HIV treatment and transmit HIV through the use of unsterilized needles and razors for traditional skin pricking or cutting practices [19]. Many PLHIV linger in THs’ offices and present late at antiretroviral treatment clinics after developing severe HIV symptoms [20]. When THs are sufficiently informed about HIV infection, they will promptly refer patients with HIV to ART clinics so that they can begin treatment as soon as possible. Furthermore, they will not spread infections if they follow good infection control practices. THs will support HIV prevention and treatment programs in this way. Few studies on THs’ HIV-related knowledge and infection control practices have been conducted in Bukavu.

In DRC, the Ministry of Health (MOH) has a National Program for the Promotion of Traditional Medicine and Medicinal Plants (PNMT/PM). The practice of traditional medicine (TM) is governed by ministerial decree n°1250/CAB/MIN/S/CJ/KIZ/62/2002 dated 10/25/2002. Before practicing, the TH must have the following documents: certificate of registration as a TH, attestation of affiliation to an association of THs, traditional healing art license, and authorization to open a TH’s office, the only place to practice TM; issued and regularly renewed under the conditions and by the competent authorities [21]. 

The objective of the current study was to assess knowledge of THs about HIV and infection control practices of THs in Bukavu City, South Kivu province in the Democratic Republic of the Congo.

## Methods

### Study design and setting

A cross-sectional study using quantitative approach was conducted from March to June 2023 among THs in the health zones of Bukavu namely Ibanda Health Zone, Kadutu Health Zone, and Bagira Health Zone. In this quantitative approach, a combination of two tools (a pre-established structured interview assisted by a questionnaire and an observational checklist) was used. The THs in South Kivu province are managed by the Provincial Coordination Office of Traditional Medicine and Medicinal Plants. The Coordination Office is located in Bukavu City, in the building of the South Kivu Provincial Health Division. These THs work in the three health zones of Bukavu City.

### Study sampling

All Traditional Healing Organization members of Bukavu City, who received PLHIV in health consultations during the study period (71 THs), were included in the study. The inclusion criteria were to be a TH recognized by the South Kivu Provincial Coordination of Traditional Medicine and Medicinal Plants, to be a TH working in one of the three communes of the city of Bukavu, to have freely accepted to participate in the study, and to treat a suspected or confirmed case of HIV infection. A session was programmed to administer previous information. The information provided related to the identity and contact details of the researchers, the aims of the research, and whether data collection was compulsory or optional.

### Data collection

A predesigned structured interviewer-assisted questionnaire was used to collect data about personal and demographic characteristics including: age, gender, level of education, religious affiliation, ethnicity, residence, history of HIV and AIDS training, and formal training in managing HIV and AIDS.

The brief HIV knowledge questionnaire (18-item version) developed by Carey and Schroder [22] was used to measure and assess the THs’ HIV-related knowledge. This tool is composed of 18 questions related to HIV/AIDS, modes of transmission, and prevention. For each of the 18 True/False, HIV- related questions, a score of 1 was assigned to each ‘correct’ answer, while a score of 0 was given to each “incorrect answer”. Five items (no. 1, 4, 11, 14, 17) are true statements, while the remaining 13 items are false. Assessments were based on the analysis of the sum of these scores, which have a possible range of 0 to 18, whereby higher scores indicated greater knowledge about HIV. Knowledge levels were classified as “poor” for those who scored 50% and below, “fair” for those who scored 51–74%, and “good” for those who scored 75% and above.

An observational checklist was used to collect data about infection control practices of THs. The items observed included hand washing practice, incision practice, reusing razor blades and needles, enema practice, using gloves when carrying out incisions, presence of protective clothing, supply of gloves, and availability of personal protective equipment (PPE) and containers to keep used blades and needles. This form has been developed by the authors and has received the opinion of three experts in public health for its suitability. The observations of the infection control practices were carried out three times for the same TH on different days. The total number of observations was 213. The duration of the observations depended on the time devoted to consultations and care administration. The observer began the observation when the TH started work and ended it when the TH had no more patients to care for. During observations, the relationships between observers and THs were neither detached nor engaged. The observers were somewhere between the two extremes of the spectrum of totally detached and totally engaged, i.e., they had different attitudes to those of the THs involved. The observers took a step back and noted the results of their observations without commenting on them. The participants were not aware that observations were being made, so they couldn’t act consciously. To improve the reliability and validity of our observations, we have used multiple independent researchers to observe and code their notes. We thought about coding responses as yes or no. Although observational research is generally associated with qualitative methods, we have always coded responses for behaviors, participant statements, and the presence or absence of something in order to get an idea of the frequency of THs’ attitudes and practices. To determine the score ranges, we used the pseudo-qualitative scale, which consists of determining regular and non-regular interval classes as follows: [0 to 20 points [or (˂ 50%), [20 to 30 points [or (50–74%), and [30 to 40 points [or (75–100%). Practice levels were classified as “poor” for those who scored 50% and below, “fair” for those who scored 51–74%, and “good” for those who scored 75% and above [23]. 

### Data management and analysis

Data completeness was checked during the data collection process. Data were entered in KoboCollect, cleaned, and coded in Microsoft Excel. The data were analyzed using Epi Info 3.5.1 and the Statistical Package for the Social Sciences (SPSS) version 16. The mean and standard deviation (SD) or median and interquartile range (IQR) were used to summarize the quantitative variables, depending upon the data distribution. Categorical variables were summarized using the frequency and percent. The independent-samples T-test and the one-way analysis of variance (ANOVA) test, were used to compare the means. Predictors of knowledge and infection control practices were identified using multivariable linear regression. Personal and demographic characteristics were the independent variables. Statistical significance was defined as a p-value < 0.05.

### Ethical considerations

The authors sought the approval of the Ethics Committee of the High Institute of Public Health, Alexandria University, Egypt (IRB N°: 00013692) for conduction of the study, and complied with International Guidelines for Research Ethics. We have followed all the procedures of the Declaration of Helsinki on medical research involving human subjects. Informed written consent was obtained from the study participants, after an explanation of the purposes and benefits of research. Anonymity and confidentiality were guaranteed and maintained. All participants were free to choose whether or not to take part in the study. The essential administrative and preliminary communications with the health authorities were carried out to rationalize the execution of the study. Authorization to conduct the study was granted by the Provincial Directorate of the National Health Ethics Committee (CNES/SK: 001-4125/001-113/2019) and by the Competent Authority of the Provincial Health Division of South Kivu (N°003/CD/DPS-SK/2019 and N°002/CD-DPS-SK/2022).

## Results

The mean age of the THs was 49.2 ± 11.2 years, and the majority (63.4%) were aged 40 to < 60 years. The majority of THs were males (88.7%), with a male-to-female ratio of 7.9. Regarding education, almost half of the THs (54.9%) had completed secondary education. Catholic and Protestant Christians were the majority among those surveyed (29.6% and 42.3%, respectively). The *Shi* and *Lega* tribes constituted 52.1% and 32.4% respectively. The THs who took part in the study were from Ibanda health zone (38.0%), Kadutu health zone (35.2%), and Bagira health zone (26.8%). More than two thirds of them (67.6%) had not received any training in taking care of patients with HIV/AIDS infection. Among those who had been trained, 82.5% had received formal training.

Most participants knew that a person cannot get HIV by sharing a glass of water with someone who has HIV (84.5%) and that having sex with more than one partner can increase a person’s chance of being infected with HIV (87.3%). On the other hand, less than 30% of participants acknowledged that a natural skin condom is no more effective against HIV than a latex condom and that coughing and sneezing do not transmit HIV. Only 8.5% of THs knew that coughing and sneezing do not spread the virus. The percentages of correct answers for the other items ranged from 30 to 80%.

**Fig. 1:**
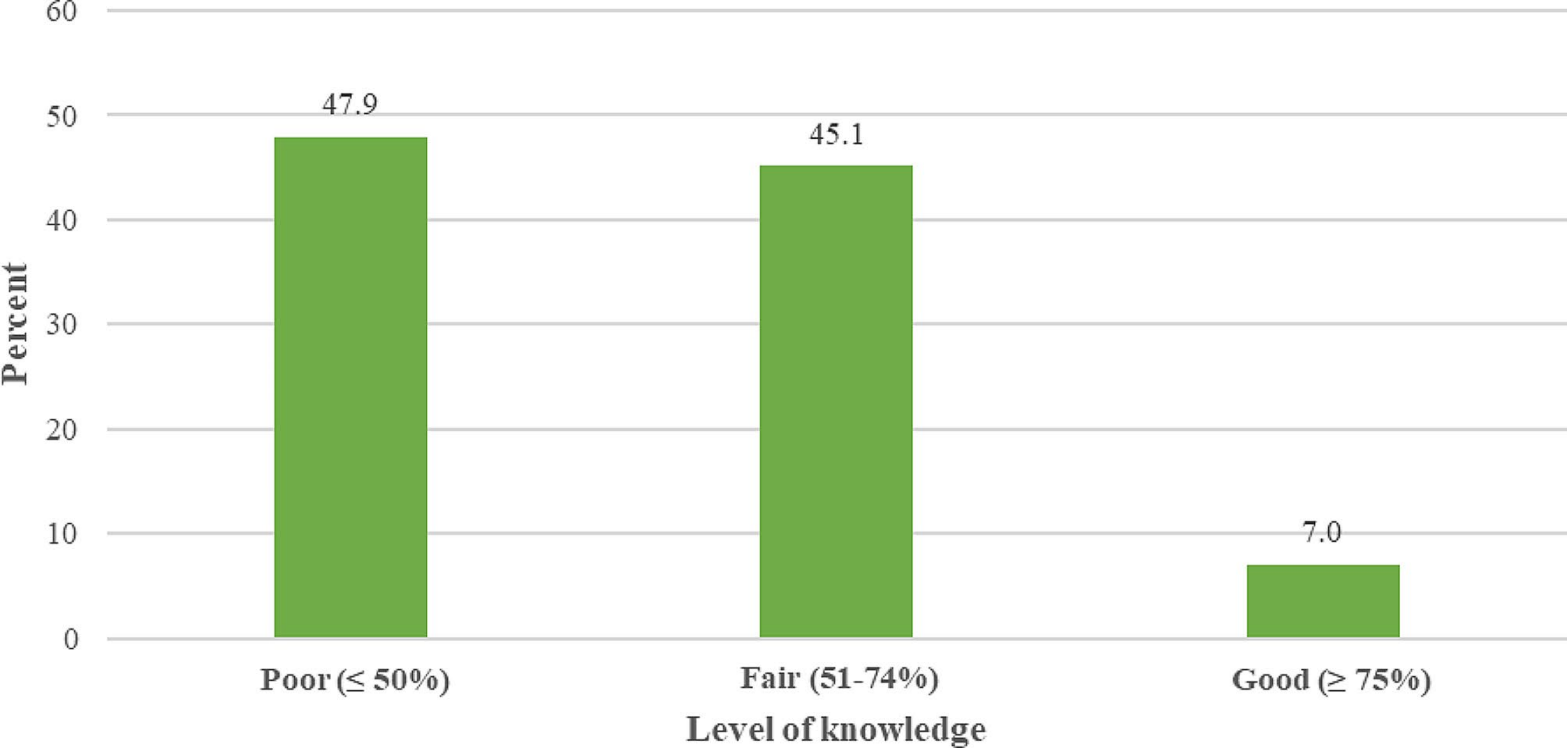
Distribution of THs according to their level of knowledge regarding HIV/AIDS (Bukavu, 2023)

Overall, 47.9% of study participants had poor knowledge about HIV/AIDS infection, 45.1% of them had fair knowledge and only 7.0% had good knowledge (Table 1; Fig. 1). The knowledge score ranged between 5.5 and 88.9 points with a mean of 53.1 ± 17.7 points.

**Table 1:** Distribution of THs according to their level of knowledge regarding HIV/AIDS (Bukavu, 2023)

Level of knowledge	No. (%)
	**(** *n* ** = 71)**
Poor (≤ 50%)	34 (47.9)
Fair (51–74%)	32 (45.1)
Good (≥ 75%)	5 (7.0)
Min – Max	5.6–88.9
Mean ± SD	53.1 ± 17.7

SD, standard deviation

Table 2 shows the availability of infection control resources at the THs’ offices. Less than two thirds of the THs (64.8%) had soap and water in their offices, and a waste bin dedicated to the disposal of medical waste (63.4%). Safety boxes for disposing of used blades and needles were present in more than one third (36.6%) of THs’ offices. Nearly a quarter of THs’ offices had a supply of gloves (25.4%) and masks (23.9%) in the treatment room. Less than a tenth of THs (8.5%) had face shields in their offices, and bloodstains could be seen on furniture or floor of their offices (9.9%).

**Table 2:** Distribution of traditional healers’ offices according to the availability of infection control-related resources (Bukavu, 2023)

Available resources#	No. (%) *n* = 71
Gloves	18 (25.4)
Masks	17 (23.9)
Face shields	6 (8.5)
Waste bin dedicated for disposal of medical waste	45 (63.4)
Safety boxes to dispose of used blades and needles	26 (36.6)
Soap and water	46 (64.8)
Blood stains on furniture or floor	7 (9.9)

#Responses are not mutually exclusive

**Fig. 2:**
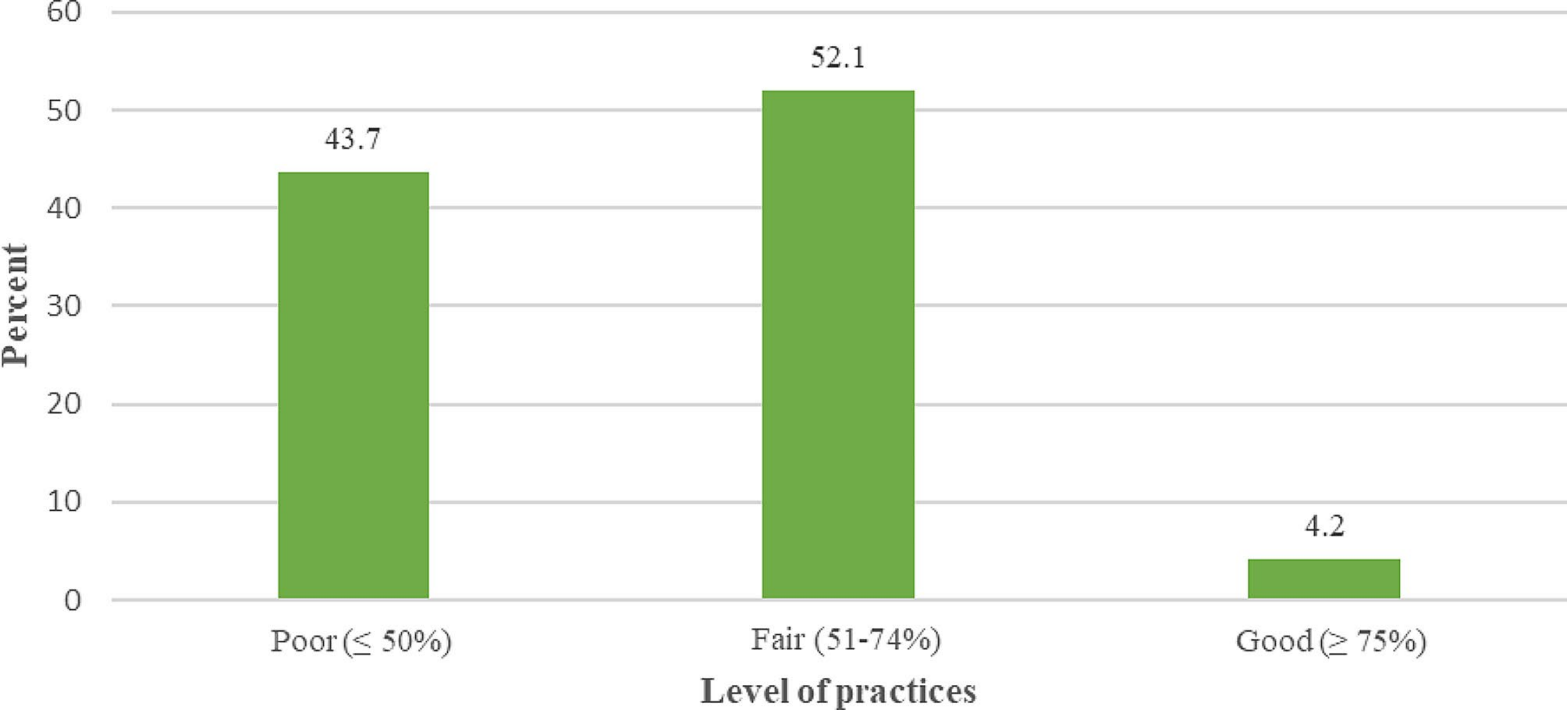
Distribution of infection control observations of THs according to the level of practice (Bukavu, 2023)

In the majority of observations, THs complied with no reuse of needles (87.3%), razor blades (74.6%), and gloves (74.6%). Two practices were complied with in 50–70% of observations: correct hand-washing technique before patient care and no scarification. Five infection control practices; namely wearing gloves when making scarification, using safety boxes to dispose used blades/needles, wearing medical gowns during care, using sterile devices while practicing enema, and wearing a mask during care were the most challenging to implement (THs complied with these practices in less than 50% of observations). Wearing face shields during care was the most difficult practice to complete (only 5.6% of observations). Generally, 43.7% of THs had poor infection control practices, 52.1% of THs had fair practices, and only 4.2% of participants had good practices (Table 3; Fig. 2). TH practice scores ranged from 9.1 to 81.8 points with a mean of 51.9 ± 15.9.

**Table 3:** Distribution of infection control observations of THs according to the level of practice (Bukavu, 2023)

Level of practice	No. (%)
	*n* = 213
Poor (≤ 50%)	93 (43.7)
Fair (51–74%)	111 (52.1)
Good (≥ 75%)	9 (4.2)
Min – Max	9.1–81.8
Mean ± SD	51.9 ± 15.9

SD, standard deviation

Table 4 compares the mean scores for HIV-related knowledge and infection control practices of THs according to their personal and demographic characteristics. The mean knowledge score of THs who had training in taking care of HIV/AIDS (10.6 ± 3.0) was slightly higher than the mean knowledge score of participants who did not have training in taking care of PLHIV (9.0 ± 3.2). No statistically significant differences were observed between participants’ mean knowledge scores for the majority of the personal and demographic characteristics studied (*p* > 0.05). The average infection control practice score of THs with a university degree (6.3 ± 1.9) was slightly higher than that of participants with up to secondary education (5.8 ± 1.7), but without showing any statistically significant difference. THs who had received training in taking care of HIV/AIDS (6.4 ± 1.6) had significantly (*p* = 0.025) higher good practices than those who had not received training in taking care of HIV/AIDS (5.4 ± 1.8). The other characteristics did not show any statistically significant difference in the mean practice scores.

**Table 4:** Comparison between the personal and demographic characteristics of THs and their HIV knowledge and infection control practice scores (Bukavu, 2023)

Characteristics	*N*	HIV-Knowledge score		T / F-test *p*-value	HIV-Practice score		T / F-test *p*-value
		Mean	SD		Mean	SD	
Age in years				0.786			0.956
20 –	16	9.1	3.0		5.8	1.4	
40 –	45	9.6	3.2		5.7	1.9	
60+	10	10.0	3.4		5.6	1.8	
Gender				0.944			0.634
Male	63	9.5	3.2		5.7	1.8	
Female	8	9.6	2.9		6.0	1.4	
Level of education				0.135			0.406
Illiterate	11	8.9	2.7		5.0	1.7	
Primary	10	7.9	3.6		5.6	1.6	
Secondary	39	9.7	3.2		5.8	1.7	
University	11	11.0	2.6		6.3	1.9	
Religion				0.429			0.336
Protestant Christian	30	9.8	3.2		5.7	1.6	
Catholic Christian	21	10.1	2.7		6.0	1.7	
Muslim	5	7.8	2.9		4.4	2.1	
Others (Brahanamists & J’s W)	15	8.9	3.8		5.9	2.1	
Tribe				0.134			0.350
*Shi*	37	9.9	2.7		6.0	1.6	
*Lega*	23	9.9	3.0		5.4	1.8	
*Havu*	4	6.7	3.9		6.2	1.5	
Others (*Bembe, Fuliru, Nande*, …)	7	8.2	4.4		5.0	2.3	
Residence				0.071			0.535
Ibanda	27	8.6	2.8		6.0	1.8	
Kadutu	25	9.2	3.4		5.7	1.7	
Bagira	19	10.7	3.0		5.4	1.9	
Training in taking care of HIV/AIDS 0.052							0.025*
Yes	23	10.6	3.0		6.4	1.6	
No	48	9.0	3.2		5.4	1.8	

J’s W, Jehovah’s Witnesses; T-test, independent-samples T test; F test, One-way ANOVA; SD, standard deviation; *Significant (*p* < 0.05)

Results of multiple linear regression analysis are summarized in Table 5. None of the personal and demographic variables studied were significant predictors for THs knowledge about HIV/AIDS (*p* > 0.05). In terms of practices, two variables were significant predictors for infection control practices; namely living in Ibanda and receiving training in taking care of HIV/AIDS.

**Table 5:** Multivariable linear regression analysis of the predictors of HIV knowledge and infection control practices among THs (Bukavu, 2023)

Characteristics	HIV-KQ-18 score				HIV-Practices score			
	Coefficient	Sdt Error	F-test	*p*	Coefficient	Sdt Error	F-test	*p*
Age in years	0.578	0.643	0.809	0.372	0.000	0.349	0.000	0.999
Gender	-0.606	1.259	0.232	0.632	-0.825	1.682	1.462	0.231
Level of education	0.444	0.43	1.064	0.306	0.185	0.233	0.628	0.431
Religion	-0.317	0.377	0.711	0.402	-0.039	0.204	0.037	0.849
Tribe	-0.525	0.375	1.956	0.167	-0.176	0.203	0.750	0.390
Residence	-0.703	0.544	1.672	0.201	-0.624	0.295	4.482	0.038*
Training on HIV	1.566	0.909	2.969	0.090	1.336	0.492	7.365	0.008*
Correlation coefficient	r^2^ = 0.15				**r**^**2**^ **= 0.18**			

* Significant (*p* < 0.05); Sdt Error, standard error; F-test, one way ANOVA

## Discussion

Most patients with HIV use traditional medicine concurrently with modern medicine. Other PLHIVs would rather visit a TH than waste time traveling to the medical facility. THs who do not have sufficient knowledge that HIV/AIDS infection can both transmit HIV using non-sterile equipment and delaying referral of PLHIV to antiretroviral treatment clinics for proper care and treatment. Prior to any intervention, knowledge, attitude, and practice studies are very important tools for assessing the extent to which individuals or communities are ready to adopt risk-free behaviors [24]. 

Regarding THs’ level of knowledge about HIV/AIDS infection, the results of the present study indicate that almost half of the participants (47.9%) had a low level of knowledge about HIV/AIDS infection. These results are similar to those of a study conducted in Mozambique in 2013, where THs had a relatively low level of knowledge about HIV/AIDS, but those who had received prior training on HIV/AIDS performed better than those who had not [25]. The results of our study are also in agreement with those of another study conducted by Sphiwe M. in 2015 in Botswana [24]. 

Regarding infection control practices, the results of this study show that the practices were poor for almost half of the THs surveyed, with an average practice score of 51.9 ± 15.9%. These results are similar to those of a study carried out in the Zambézia province of Mozambique. In this study, the authors found that the majority of therapists were repeatedly exposed to patients’ blood. THs gave injections and used a new razor an average of three times. What’s more, they almost never used gloves during these procedures [26]. Different findings were also reported by other studies conducted by Audet et al. and Ndou-Mammbona, in South Africa. Audet et al. found a higher score level of poor practices compared to our study. In their study, almost all traditional healers routinely performed minimally invasive skin incisions that could expose them to patients’ blood. They found that THs were not trained in infection control and lacked PPE [27]. Ndou-Mammbona’s study reveals that some traditional healings have had a negative impact on the management of HIV and AIDS. The following negative aspects had been reported: the promise of cure, denial of the presence of HIV, telling people to stop treatment, use of incisions, killing the virus, using emetics and enemas to cleanse the person from HIV, having an ancestral calling, and believing that they are bewitched [28]. Poor practices could be explained by the fact that the majority of THs did not have the necessary resources, such as face shields, masks, gloves and safety boxes for disposing of razor blades and used needles. Blood stains on the furniture and/or floors of treatment rooms were observed at some THs’ offices. In addition to the unavailability of infection-control resources, some THs practiced poor infection control practices that could expose patients to transmissible infections, for example failure to wear protective equipment during treatment, reuse of certain single-use PPE, and using unsterile devices for the purpose of enema and scarification practices. Non-compliance with infection control practices could be attributed to ignorance or low levels of education, negligence, and lack of financial resources. Comparing the mean practice scores of THs revealed that THs with a higher level of education and HIV/AIDS management training had higher mean practice scores compared to those with low level of education and without training. Results of the multivariate analysis using the linear regression model showed significant differences between the mean practice scores. Two variables were the significant predictors, namely living in Ibanda and receiving training in HIV/AIDS management.

Regardless of our scientific rigor, this study has certain limitations linked to the size of the sample (there are not many officially recognized traditional practitioners in the province) but also to the lack of recent previous studies on the subject (the majority of previous studies were dated from 2010 to 2020). For future research, a longitudinal study could be conducted to assess THs’ HIV knowledge, attitudes, and infection control practices using a large population in order to generalize the findings.

## Conclusion and recommendations

In conclusion, THs had inadequate knowledge about HIV/AIDS infection and poor infection control practices. Regular formal and standardized HIV/AIDS infection training for all THs, to improve their knowledge on HIV/AIDS and their infection prevention and control practices while treating patients in their clinics is recommended. Province health authority must also supervise infection control practices in THs’ offices, but PPE’s support for THs is also needed in terms of protection measures.

### Electronic supplementary material

Below is the link to the electronic supplementary material.

Supplementary Material 1

## Data Availability

No datasets were generated or analysed during the current study.

## Abbreviations

HIV: Human Immunodeficiency Virus
AIDS: Acquired Immunodeficiency Syndrome
PLHIV: People Living with Human Immunodeficiency Virus
THs: Traditional Healers
ART: Antiretroviral Therapy
PPE: Personal Protective Equipment
