# Supplementary material for: Traditional healers’ knowledge and infection control practices related to HIV in Bukavu City, Democratic Republic of the Congo
Source: BMC Public Health. 2024 May 27;24:1403. doi: 10.1186/s12889-024-18941-9 (PMC11129489; doi:10.1186/s12889-024-18941-9)
Supplement: Supplementary file 1 — Supplementary Material 1 [file 12889_2024_18941_MOESM1_ESM.docx]

**Supplementary Table (1): Distribution of THs according to their personal and socio-demographic characteristics (Bukavu, 2023)**

| Personal and socio-demographic characteristics | No. (n=71) | % |
| --- | --- | --- |
| Age in years |  |  |
| 20 – | 16 | 22.5 |
| 40 – | 45 | 63.4 |
| 60 + | 10 | 14.1 |
| Min – Max | 24 – 73 | |
| Mean ± SD | 49.2 ± 11.2 | |
| Gender |  |  |
| Male | 63 | 88.7 |
| Female | 8 | 11.3 |
| Level of education |  |  |
| Illiterate | 11 | 15.5 |
| Primary | 10 | 14.1 |
| Secondary | 39 | 54.9 |
| University | 11 | 15.5 |
| Religion |  |  |
| Protestant Christians | 30 | 42.3 |
| Catholic Christians | 21 | 29.6 |
| Muslims | 5 | 7.0 |
| Others (Brahanamists and Jehovah’s Witnesses) | 15 | 21.1 |
| Tribe |  |  |
| *Shi* | 37 | 52.1 |
| *Lega* | 23 | 32.4 |
| *Havu* | 4 | 5.6 |
| Others (*Bembe, Fuliru, Nande, Songe, Tembo, Luba*) | 7 | 9.9 |
| Health zone |  |  |
| Ibanda | 27 | 38.0 |
| Kadutu | 25 | 35.2 |
| Bagira | 19 | 26.8 |
| Training in taking care of patients with HIV/AIDS infection | |  |
| Yes | 23 | 32.4 |
| No | 48 | 67.6 |
| Formal training (n=23)^a^ |  |  |
| Yes | 19 | 82.6 |
| No | 4 | 17.4 |

^a^ % calculated among traditional healers who reported being trained on taking care of patients with HIV/AIDS; SD, standard deviation.

**Supplementary Table (2): Distribution of THs according to their knowledge related to HIV/AIDS (Bukavu, 2023)**

| HIV/AIDS-related knowledge | No. (%) |
| --- | --- |
|  | **(n=71)** |
| Coughing and sneezing do not spread HIV (correct) | 6 (8.5) |
| A person can get HIV by sharing a glass of water with someone who has HIV (correct) | 60 (84.5) |
| Pulling out the penis before a man climaxes/cums keeps a woman from getting HIV during sex (correct) | 45 (63.4) |
| A woman can get HIV if she has anal sex with a man (correct) | 47 (66.2) |
| Showering, or washing one’s genitals/private parts, after sex keeps a person from getting HIV (correct) | 50 (70.4) |
| All pregnant women infected with HIV quickly show serious signs of being infected (correct) | 37 (52.1) |
| People who have been infected with HIV quickly show serious signs of being infected (correct) | 54 (76.1) |
| There is a vaccine that can stop adults from getting HIV (correct) | 39 (54.9) |
| People are likely to get HIV by deep kissing, putting their tongue in their partner’s mouth, if their partner has HIV (correct) | 33 (46.5) |
| A woman cannot get HIV if she has sex during her period (correct) | 23 (32.4) |
| There is a female condom that can help decrease a woman’s chance of getting HIV (correct) | 29 (40.8) |
| A natural skin condom works better against HIV than a latex condom (correct) | 10 (14.1) |
| A person will not get HIV if she or he is taking antibiotics (correct) | 36 (50.7) |
| Having sex with more than one partner can increase a person’s chance of being infected with HIV (correct) | 62 (87.3) |
| Taking a test for HIV one week after having sex will tell a person if she or he has HIV (correct) | 35 (49.3) |
| A person can get HIV by sitting in a hot tub or a swimming pool with a person who has HIV (correct) | 51 (71.8) |
| A person can get HIV from oral sex (correct) | 36 (50.7) |
| Using Vaseline or baby oil with condoms lowers the chance of getting HIV (correct) | 25 (35.2) |

**Supplementary Table (3): Distribution of infection control observations of THs (Bukavu, 2023)**

| Compliance with infection control practices# | No. (%) |
| --- | --- |
|  | **(n=213)** |
| Correct hand washing technique before patient care | 129 (60.6) |
| No scarification | 123 (57.7) |
| Wearing gloves when making scarification | 54 (37.5) |
| No reuse of gloves | 159 (74.6) |
| No reuse of razor blades | 159 (74.6) |
| No reuse of needles | 186 (87.3) |
| Using safety boxes to dispose of used blades/needles | 72 (33.8) |
| Using unsterile devices for the purpose of enema | 63 (29.6) |
| Wearing medical gowns during care | 72 (33.8) |
| Wearing masks during care | 48 (22.5) |
| Wearing face shields during care | 12 (5.6) |

#Responses are not mutually exclusive
